# Coplanar Polychlorinated Biphenyls Impair Glucose Homeostasis in Lean C57BL/6 Mice and Mitigate Beneficial Effects of Weight Loss on Glucose Homeostasis in Obese Mice

**DOI:** 10.1289/ehp.1205421

**Published:** 2012-10-24

**Authors:** Nicki A. Baker, Michael Karounos, Victoria English, Jun Fang, Yinan Wei, Arnold Stromberg, Manjula Sunkara, Andrew J. Morris, Hollie I. Swanson, Lisa A. Cassis

**Affiliations:** 1 Graduate Center for Nutritional Sciences; 2 Department of Chemistry; 3 Department of Statistics; 4 Division of Cardiovascular Medicine, and; 5 Department of Molecular and Biomedical Pharmacology, University of Kentucky, Lexington, Kentucky, USA

**Keywords:** adipose, diabetes, glucose tolerance, polychlorinated biphenyl

## Abstract

Background: Previous studies demonstrated that coplanar polychlorinated biphenyls (PCBs) promote proinflammatory gene expression in adipocytes. PCBs are highly lipophilic and accumulate in adipose tissue, a site of insulin resistance in persons with type 2 diabetes.

Objectives: We investigated the *in vitro* and *in vivo* effects of coplanar PCBs on adipose expression of tumor necrosis factor α (TNF-α) and on glucose and insulin homeostasis in lean and obese mice.

Methods: We quantified glucose and insulin tolerance, as well as TNF-α levels, in liver, muscle, and adipose tissue of male C57BL/6 mice administered vehicle, PCB-77, or PCB-126 and fed a low fat (LF) diet. Another group of mice administered vehicle or PCB-77 were fed a high fat (HF) diet for 12 weeks; the diet was then switched from HF to LF for 4 weeks to induce weight loss. We quantified glucose and insulin tolerance and adipose TNF-α expression in these mice. In addition, we used *in vitro* and *in vivo* studies to quantify aryl hydrocarbon receptor (AhR)-dependent effects of PCB-77 on parameters of glucose homeostasis.

Results: Treatment with coplanar PCBs resulted in sustained impairment of glucose and insulin tolerance in mice fed the LF diet. In PCB-77–treated mice, TNF-α expression was increased in adipose tissue but not in liver or muscle. PCB-77 levels were strikingly higher in adipose tissue than in liver or serum. Antagonism of AhR abolished both *in vitro* and *in vivo* effects of PCB-77. In obese mice, PCB-77 had no effect on glucose homeostasis, but glucose homeostasis was impaired after weight loss.

Conclusions: Coplanar PCBs impaired glucose homeostasis in lean mice and in obese mice following weight loss. Adipose-specific elevations in TNF-α expression by PCBs may contribute to impaired glucose homeostasis.

Type 2 diabetes (T2D) affects 300 million people, and diabetes prevalence is anticipated to double world-wide over the next 20 years (Shaw et al. 2010). Recent epidemiological studies suggested that exposure to low concentrations of polychlorinated biphenyls (PCBs) similar to current exposure levels in humans increases diabetes risk (Lee et al. 2010). Multiple cross-sectional analyses of National Health and Nutrition Examination Survey (NHANES) cohorts from 1999–2006 found concentrations of PCB-170 in urine or blood with an adjusted odds ratio of 4.5 for T2D, predicting up to 15% risk (Patel et al. 2010). Remarkably, this increased risk appeared in individuals who were not overweight or obese (Carpenter 2008). Similar observations linking PCB exposures to T2D have been reported in other populations (Codru et al. 2007; Wang et al. 2008). Recently, a significant association between elevated PCB levels and diabetes was found in the Anniston Community Health Survey (Silverstone et al. 2012). Collectively, accumulating evidence supports a link between PCB exposure levels and the development of diabetes, but mechanisms linking PCBs to diabetes are largely unknown.

Because of their lipophilicity, PCBs accumulate in lipid stores of adipose tissue (Kodavanti et al. 1998), suggesting that adipocytes experience continuous low-grade exposures to PCBs. Extension of these findings to the setting of obesity suggests that the total body burden of PCBs would increase in obese subjects because of the inherent lipophilicity of these compounds. Recent studies demonstrated an inverse relationship between serum levels of several PCBs and body mass index, suggesting that sequestration of PCBs in adipose tissue is enhanced by obesity (Dirinck et al. 2011). It is unclear whether this enhanced sequestration limits effects of PCBs systemically. Given the epidemic of obesity and T2D in the United States, a large percentage of the population strives to lose weight. Weight loss, through dietary restriction or increased physical activity, typically reduces adipose tissue mass through liberation of lipid stores (Walford et al. 1999). Lim et al. (2011) reported that plasma concentrations of organochlorines were increased and significantly correlated to reductions in body mass index in obese subjects undergoing a weight-loss program. Further, Pelletier et al. (2002) found that plasma concentrations of 13 of 17 organochlorines in serum increased in obese subjects experiencing weight loss. The consequences of increased liberation of PCBs following weight loss in obese subjects with T2D are unknown.

Previous studies in our laboratory demonstrated that low concentrations of coplanar PCB-77 (3,3´,4,4´-tetrachlorobiphenyl), a ligand of the aryl hydrocarbon receptor (AhR), promoted adipocyte differentiation and the production of proinflammatory adipokines (Arsenescu et al. 2008). Recent studies showed that PCB-126 (3,3´,4,4´,5-pentachlorobiphenyl), a coplanar AhR ligand, promoted inflammatory gene expression in human preadipocytes and adipocytes, and also increased inflammatory gene expression in adipose tissue from wild-type, but not AhR-deficient, mice (Kim et al. 2012). Among several inflammatory cytokines stimulated by coplanar PCBs in adipocytes (Arsenescu et al. 2008; Kim et al. 2012), tumor necrosis α (TNF-α) is a recognized contributor to insulin resistance (Hotamisligil et al. 1996; Ventre et al. 1997). TNF-α promotes insulin resistance through downstream alternative phosphorylation of the insulin receptor (IR) docking protein, insulin receptor substrate-1 (IRS-1) (Peraldi et al. 1996), which prevents phosphorylation of protein kinase B (Akt) (Thong et al. 2005) and impairs transport of glucose transporter type 4 vesicles in skeletal muscle and adipose tissue to the plasma membrane (Peraldi and Spiegelman 1998). TNF-α can indirectly contribute to adipocyte insulin resistance by promoting lipolysis (Cawthorn and Sethi 2008) and/or by inhibiting differentiation (Torti et al. 1989).

In the present study, we defined dose-dependent effects of coplanar PCBs on glucose homeostasis in lean mice. To define mechanisms for PCB-induced impairment of glucose and insulin tolerance, we quantified expression levels of TNF-α in organs contributing to insulin resistance. Moreover, we defined the sustainability of PCB-induced impairment of glucose homeostasis in lean mice in reference to organ and serum concentrations of PCBs. To delineate the role of AhR in effects of PCBs, we examined *in vitro* and *in vivo* effects of an AhR antagonist on parameters of glucose homeostasis. Because T2D is frequently associated with obesity, we examined effects of a coplanar PCB on glucose homeostasis in obese mice. In addition, because PCBs redistribute from adipose tissue to the circulation during weight loss, we determined effects of previous PCB exposures on glucose homeostasis in obese mice experiencing weight loss.

## Materials and Methods

*Chemicals.* We purchased PCB-77 and PCB-126 from AccuStandard Inc. (New Haven, CT). 2-Methyl-2H-pyrazole-3-carboxylic acid (2-methyl-4-*o*-tolylazo-phenyl-amide; CH-223191) was a generous gift from H. Swanson (University of Kentucky, Lexington, KY).

*Animal treatments and sample collection.* All experimental procedures met the approval of the Animal Care and Use Committee of the University of Kentucky. We treated animals humanely and with regard for alleviation of suffering. Male C57BL/6 mice (2 months of age; The Jackson Laboratory, Bar Harbor, ME) were given *ad libitum* access to food and water and housed in a pathogen-free environment. Initial studies examined glucose and insulin tolerance in mice administered vehicle (tocopherol-stripped safflower oil), PCB-77 (2.5, 50, or 248 mg/kg; by oral gavage given as two separate doses over 2 weeks; *n* = 10 mice/group), or PCB-126 (0.3, 1.6, or 3.3 mg/kg once by oral gavage; *n* = 10 mice/group). Mice in dose–response studies were fed a low fat diet (LF; 10% kcal as fat; Research Diets Inc., New Brunswick, NJ).

To define the sustainability of PCB effects, we performed temporal studies in mice fed a LF diet. Mice were administered vehicle or PCB-77 (50 mg/kg by oral gavage) once in week 1 and once in week 2. For mice examined at later time points (12 weeks), a second set of treatment by oral gavages was administered in weeks 9 and 10. Mice in each treatment group (vehicle or PCB-77) were examined at weeks 2, 4, or 12 after onset of feeding with LF diet. Body weights were quantified weekly in all studies. At the study end point, mice were anesthetized (ketamine/xylazine, 10/100 mg/kg, by intraperitoneal (ip) injection] for exsanguination and tissue harvest (liver, soleus muscle, and visceral adipose). A subset of mice (*n* = 5/group) in each treatment group were administered insulin [10 U/kg (Kienesberger et al. 2009)] 10 min before exsanguination to elicit insulin signaling pathways.

For studies examining obesity and weight loss, male C57BL/6 mice (2 months of age; *n* = 10/group) were fed a high fat diet (HF; 60% kcal as fat; Research Diets Inc.) for 12 weeks and administered vehicle or PCB-77 (50 mg/kg by oral gavage) at weeks 1, 2, 9, and 10. Body weight was quantified weekly. At 12 weeks of HF feeding, a subset of mice (*n* = 3/group) was anesthetized for exsanguination and tissue harvest. The remaining mice in each treatment group were then fed the LF diet for 4 weeks to induce weight loss.

To determine whether *in vivo* effects were AhR-mediated, we fed male C57BL/6 mice (2 months of age; *n* = 7/group) the LF diet and orally gavaged them with vehicle or CH-223191 daily (10 mg/kg/day) (Kim et al. 2006; Choi et al. 2012) starting 1 week before administration of vehicle or PCB-77 and through the remainder of the study. After 1 week of pretreatment with CH-233191, mice were administered vehicle or PCB-77 (50 mg/kg by oral gavage, given as two separate doses in weeks 1 and 2). Within 48 hr after the last dose of vehicle/PCB-77, we performed glucose and insulin tolerance tests.

*Glucose tolerance tests (GTT) and insulin tolerance tests (ITT).* Mice were fasted for 6 hr or 4 hr prior to quantification of GTT or ITT, respectively. Blood collected from the tail vein was tested for glucose concentration using a handheld glucometer (Freedom Freestyle Lite; Abbott Laboratories, Abbott Park, IL). Mice were injected with d-glucose (20% in saline, ip) or human insulin (Novolin, 0.0125 µM/g body weight, ip) and blood glucose was quantified at 0–120 min. Total area under the curve (AUC; arbitrary units), which is obtained without the presence of a baseline, calculates the area below the observed concentrations and, in comparison studies, was reported to compare favorably to the positive incremental area method for statistical analyses of glucose tolerance data (Cardoso et al. 2011).

*Quantification of PCBs.* Frozen tissue samples (epididymal adipose tissue, liver, and skeletal muscle) or mouse sera were spiked with surrogate standards (100 µL of 0.35 ng/µL PCB-166 in isooctane). After the addition of 1.0 g of diatomaceous earth, the mixture was grained into fine powders and transferred into an extraction cell filled with Ottawa Sands (Thermo Fisher Scientific, Pittsburgh, PA). Extraction with hexane (30 mL) was performed using an ASE 200 accelerated solvent extractor (Dionex Corporation, Sunnyvale, CA). Lipids were removed by the addition of an equal volume of concentrated sulfuric acid. For adipose samples containing high lipid content, hexane extracts were subjected to delipidation by passing them over a Florisil SPE column, according to the manufacturer’s instructions, before acid treatment. The hexane layer containing PCBs was collected and concentrated to 2 mL using a Heidolph Synthesis1 evaporator (Heidolph, Schwabach, Germany) and then to 100 µL using a gentle stream of nitrogen. Then, 10 µL of of internal standard PCB-209 (1.0 ng/µL) was added.

Gas chromatographic analysis was performed with a gas chromatography (GC)–mass spectrometry (MS) system (Agilent 6890N GC, G2913A auto sampler, and 5975 MS detector; Agilent Technologies, Santa Clara, CA) using an HP-5MS 5% phenyl methyl siloxane column (30 m length, 0.25 mm internal diameter, 0.25 µm film thickness). The column temperature was held at 60°C for 1 min, increased to 200°C at a rate of 40°C/min, followed by a rate of 4°C/min to 280°C, and then held for 5.5 min. The injector temperature was 250°C. The carrier gas was ultrapure helium, and the makeup gas was nitrogen. PCBs were identified on the basis of their retention times relative to standards. Quantification was achieved based on calibration curves obtained using PCB standards. The recovery efficiency was calculated from the surrogates and the sample weight.

*Quantification of plasma components.* We quantified plasma insulin concentrations using a commercial ELISA (Crystal Chem, Downers Grove, IL). Plasma TNF-α and interleukin-6 (IL-6) concentrations were quantified using a commercial Milliplex MAP Mouse Serum Adipokine kit (Millipore, St. Charles, MO).

*Extraction of RNA and quantification of mRNA abundance using real-time polymerase chain reaction (PCR).* Total RNA was extracted from tissues using the SV Total RNA Isolation System kit (Promega Corporation, Madison, WI) (Arsenescu et al. 2008). RNA concentrations were quantified and cDNA was synthesized from total RNA and amplified using an iCycler (Bio-Rad, Hercules, CA) with the Perfecta SYBR Green Fastmix for iQ (20 µL; Quanta Biosciences, Gaithersburg, MD). Using the difference from 18S rRNA (reference gene) and the ΔΔCt method, we calculated the relative quantification of gene expression. The PCR reaction was 94°C for 5 min, 40 cycles at 94°C for 15 sec, 58°C or 64°C (based on tested primer efficiency) for 40 sec, 72°C for 10 min, and 100 cycles from 95°C to 45.5°C for 10 sec. Primer sequences were as follows: 18S, forward 5´AGTCGGCATCGTTTATGGTC-3´, reverse 5´-CGAAAGCATTTGCCAAGAAT-3´; *CYP1A1* (cytochrome P450 1A1), forward 5´AGTCAATCTGAGCAATGAGTTTGG-3´, reverse 5´-GGCATCCAGGGAA GAGTTAGG-3´; *F4/80* (macrophage marker), forward 5´-CTTTGGCTATGGGCTTCCAGTC-3´, reverse 5´-GCAAGGAGGACAGAGTTTATCGTG-3´; *TNF*-α, forward 5´-CCCACTCTGACCCCTTTACTC-3´, reverse 5´-TCACTGTCCCAGCATCTTGT-3´.

*Western blotting.* Tissues (liver, soleus muscle, and epididymal adipose tissue) were homogenized in mammalian protein extraction reagent (M-PER; Thermo Scientific, Rockford, IL), sonicated, and centrifuged, and supernatants were used to quantify protein. Proteins were resolved on 4–15% precast polyacrylamide gels (Bio-Rad) and transferred onto a polyvinylidene difluoride transfer membrane (GE Healthcare, Buckinghamshire, UK); nonspecific proteins were blocked for 1 hr at room temperature [5% nonfat milk in phosphate-buffered saline (PBS) in 0.1% Tween-20 for 60 min at 25°C]. Membranes were then incubated with primary antibody: rabbit anti-TNF-α (1:1,000; Novus Biologicals, Littleton, CO), mouse β-actin (1:5,000; Cell Signaling Technology, Beverly, MA) in diluted PBS overnight at 4°C. After stripping primary antibody, membranes were incubated with secondary goat anti-rabbit IgG horseradish peroxidase–linked antibody (1:5,000; Cell Signaling Technology) for 1 hr at room temperature. Protein levels were quantified using Kodak Molecular Imaging Software (Carestream Health Inc., Rochester, NY).

*Cell culture.* 3T3-L1 mouse preadipocytes, obtained from American Type Culture Collection (Manassas, VA) were cultured as described previously (Arsenescu et al. 2008). Differentiation was induced by incubating cells for 2 days with media containing insulin (0.1 μM; Sigma Chemical Co., St. Louis, MO), dexamethasone (1 μM; Sigma), and isobutylmethyl xanthine (IBMX; 0.5 mM; Sigma), and then incubating with insulin for 1 additional day. Assays (*n* = 3/group) were performed on duplicate wells of cells. Differentiated adipocytes (day 8) were incubated with vehicle [0.03% dimethyl sulfoxide (DMSO)] or PCB-77 (3.4 μM) for 24 hr (Arsenescu et al. 2008). The concentration of PCB-77 was based on the maximum serum level of PCB-77 (0.84 µg/g, converted to ≈ 3 µM) measured in experimental mice, as described above, at week 2 after administration of PCB-77 (50 mg/kg). Adipocytes from each treatment group were incubated with the AhR antagonist α-naphthoflavone (α-NF; 20 µM) for 30 min before adding vehicle or PCB-77 (3.4 μM).

*Statistical analysis.* Data are represented as mean ± SE. Total AUC and mRNA abundance data were log-transformed. We used one-way analysis of variance (ANOVA; SigmaPlot, version 12.0; Systat Software Inc., Chicago, IL) to define treatment effects of different doses of PCBs and for studies using CH-223191. In studies examining sustainability of PCB effects in lean mice, total AUC was analyzed using two-way ANOVA with the Holm-Sidak method for post hoc analysis. In studies of obese mice and mice undergoing weight loss, we performed a mixed model analysis of repeated measures and analyzed total AUC using two-way ANOVA (JMP, version 10; SAS Institute Inc., Cary, NC). Statistical significance was defined as *p* < 0.05.

## Results

**Figure 1:**
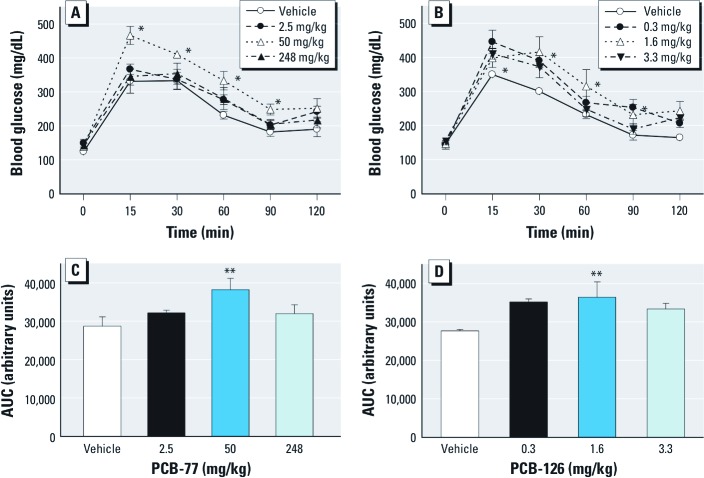
PCB-77 and PCB-126 impaired glucose tolerance in LF-fed mice. Blood glucose concentrations were examined in mice administered vehicle, PCB‑77 (2.5, 50, or 248 mg/kg; *A*), or PCB‑126 (0.3, 1.6, or 3.3 mg/kg; *B*) twice during 2 weeks, and then given a bolus of glucose 48 hr after the second dose. (*C,D*) Quantification of total AUC for data in *A* and *B*, respectively. Data are mean ± SE and represent five mice per treatment group. *p< 0.05 compared with vehicle within a time point. **p < 0.05 compared with vehicle.

*Coplanar PCBs dose-dependently impair glucose and insulin tolerance in LF-fed mice in an AhR-dependent manner.* We defined dose-dependent effects of two coplanar PCBs (PCB-77 and PCB-126)—both of which are prevalent in the environment (McFarland and Clarke 1989) and/or linked to T2D (Carpenter 2008; Everett et al. 2007)—on glucose and insulin tolerance in LF-fed mice. Forty-eight hours after the final dose, animals treated with PCB-77 (50 mg/kg) or PCB-126 (1.6 mg/kg) showed a significant increase in blood glucose concentrations, compared with controls, in response to a bolus of administered glucose (Figure 1A and B, respectively; *p* < 0.05). The total AUC for blood glucose concentrations was significantly increased in mice treated with PCB-77 or PCB-126 (Figure 1C and D, respectively; *p* < 0.05). Similarly, blood glucose concentrations in response to insulin administration were significantly increased in mice administered PCB-77 (50 mg/kg) or PCB-126 (3.3 mg/kg) [see Supplemental Material, Figure S1A and B, respectively (http://dx.doi.org/10.1289/ehp.1205421); *p* < 0.05], resulting in a significant increase in total AUC (Figure S1C and D, respectively; *p* < 0.05). Fasting plasma insulin concentrations were similar in mice administered vehicle (mean ± SE, 0.18 ± 0.10) or PCB-77 (0.19 ± 0.09 ng/ml; *p* > 0.05). On the basis of a higher prevalence of PCB-77 in food compared with PCB-126 (Hansen 1998), we used PCB-77 in further studies examining mechanisms of glucose and insulin intolerance.

To determine whether *in vivo* effects of PCB-77 are AhR-mediated, we defined effects of CH-223191, an AhR antagonist, on PCB-77–induced impairment of glucose and insulin tolerance in LF-fed mice. Administration of PCB-77 significantly impaired glucose and insulin tolerance compared with vehicle, and these effects of PCB-77 were abolished in mice administered CH-223191 [see Supplemental Material, Figure S2; *p* < 0.05 (http://dx.doi.org/10.1289/ehp.1205421)].

*PCB-77 treatment results in sustained impairment of glucose and insulin tolerance in LF-fed mice.* To define the duration of PCB-induced impairment of glucose tolerance, we administered vehicle or PCB-77 (50 mg/kg) to LF-fed (12 weeks) male C57BL/6 mice in two doses in weeks 1 and 2 of LF feeding, and then again in weeks 9 and 10 of LF feeding. Body weight was not significantly different in mice administered vehicle (mean ± SE, 27 ± 1 g) or PCB-77 (28 ± 1 g; *p* > 0.05). Glucose tolerance was significantly impaired in PCB-77–treated mice compared with controls from weeks 2 to 12 of LF feeding [see Supplemental Material, Figure S3A (http://dx.doi.org/10.1289/ehp.1205421)]. Insulin tolerance was significantly impaired in PCB-77–treated mice compared with controls on weeks 2 and 4 (see Supplemental Material, Figure S3B).

Due to their lipophilicity, PCBs accumulate in adipose tissue (Kodavanti et al. 1998). We quantified levels of PCB-77 in retroperitoneal white adipose (RPF), liver, skeletal muscle, and serum following administration of PCB-77. PCB-77 levels were undetectable in tissues or serum from vehicle-treated mice; in addition, PCB-77 was not detected in skeletal muscle from either treatment group. At week 2, PCB-77 levels in RPF (mean ± SE, 192 ± 36 µg/g) were 10 and 384 times those in liver or serum (19 ± 1 and 0.5 ± 0.2 µg/g, respectively). At week 3, PCB-77 levels in RPF (91 ± 37 µg/g) were decreased (by 53%) compared with those in week 2 (192 ± 36 µg/g) and were markedly decreased by weeks 4 and 12.

**Figure 2:**
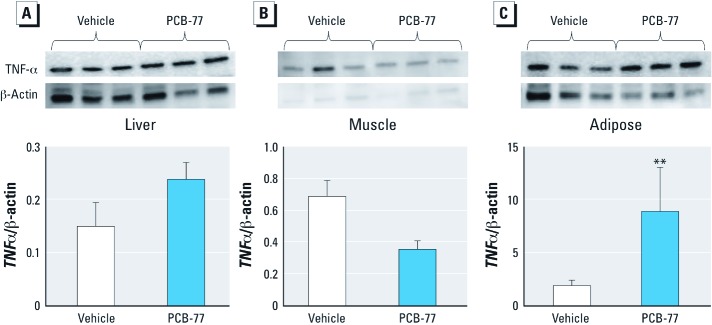
PCB-77–treated LF-fed mice had elevated TNF-α expression in adipose tissue (*C*), but not in liver (*A*) or soleus muscle (*B*). Expression levels of *TNF-*α were quantified in week 4, 2 weeks after the second dose of vehicle or PCB-77 (50 mg/kg). Data are mean ± SE and represent three mice per treatment group. **p < 0.05 compared with vehicle.

Previous studies showed that PCB-77 increased *TNF*-α mRNA abundance in 3T3-L1 adipocytes (Arsenescu et al. 2008). Thus, we contrasted effects of PCB-77 on *TNF*-α expression in adipose tissue compared with liver and skeletal muscle. As evidence of AhR activation by PCB-77, mRNA abundance of *CYP1A1*, an AhR target gene (Denison and Nagy 2003), was significantly increased in adipose tissue from PCB-77–treated mice compared with controls [see Supplemental Material, Figure S4A (http://dx.doi.org/10.1289/ehp.1205421)]. By comparison, *CYP1A1* mRNA abundance was significantly increased in livers of PCB-77–treated mice compared with controls at week 2 but not at later time points (see Supplemental Material, Figure S4B). Although *TNF*-α mRNA expression in adipose tissue of PCB-77–treated mice did not reach statistically significant levels (see Supplemental Material, Figure S5A), TNF-α protein was significantly increased at week 4 (Figure 2; *p* < 0.05). Plasma concentrations of TNF-α were significantly increased in PCB-77–treated mice compared with controls (see Supplemental Material, Figure S6A; *p* < 0.05). Plasma concentrations of IL-6 were significantly increased at week 12 in mice administered PCB-77 compared with vehicle controls (see Supplemental Material, Figure S6B; *p* < 0.05).

Infiltration of macrophages into adipose tissue has been suggested as a mechanism contributing to low-grade inflammation from obesity and the development of insulin resistance (Harford et al. 2011). Adipose tissue from mice administered PCB-77 exhibited statistically similar mRNA abundance of the macrophage marker F4/80 compared with controls [see Supplemental Material, Figure S5 (http://dx.doi.org/10.1289/ehp.1205421)].

**Figure 3:**
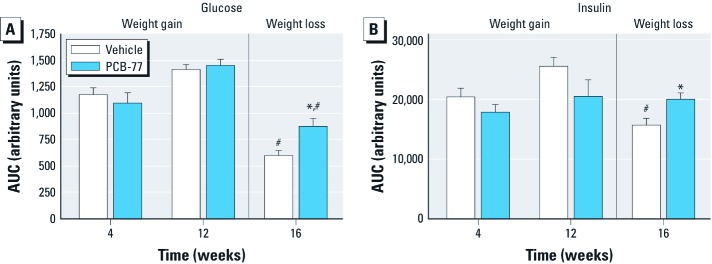
PCB-77 had no effect on glucose (*A*) or insulin (*B*) tolerance in HF-fed mice during weight gain, but impaired glucose homeostasis during weight loss. Quantification of total AUC for glucose (*A*) and insulin (*B*) tolerance in mice administered vehicle or PCB-77 (50 mg/kg, two doses during weeks 1 and 2 and two more doses during weeks 9 and 10) during weight gain (weeks 4 and 12 of HF feeding) or weight loss (week 16, after mice were switched to the LF diet). Data are mean ± SE and represent five mice per treatment group. *p< 0.05 compared with vehicle within a time point. ^#^*p *< 0.05 compared with week 12 within a treatment group.

*Effects of PCB-77 to promote glucose and insulin intolerance are lost in mice with diet-induced obesity, but manifest when obese mice lose weight.* Obesity is associated with the development of insulin resistance in T2D (DeFronzo 2004). Moreover, obesity increases the total body burden of lipophilic PCBs (Kim et al. 2011). Thus, we examined effects of PCB-77 on glucose and insulin tolerance in mice fed a HF diet for 12 weeks (weight gain phase). In addition, we examined effects of PCB-77 on glucose and insulin tolerance in HF-fed obese mice (12 weeks) that were switched to the LF diet for 4 weeks to induce weight loss (weight loss phase). During the weight gain phase, PCB-77 treatment had no significant effect on body weight in mice fed either the LF or HF diet compared with controls [see Supplemental Material, Figure S7A (http://dx.doi.org/10.1289/ehp.1205421)]. Surprisingly, PCB-77 treatment had no effect on glucose or insulin tolerance in HF-fed mice (weeks 4 or 12; Figure 3; weight gain phase). Moreover, abundance of *TNF-*α mRNA in adipose tissue was not significantly different in PCB-77–treated mice compared with controls (see Supplemental Material, Figure S8). At 12 weeks, adipose tissue levels of PCB-77 (1.8 ± 0.5 µg/g tissue, wet weight) in mice fed the HF diet were 2 times those observed in adipose tissue from LF-fed mice.

Previous studies showed that plasma concentrations of PCBs increased in obese subjects experiencing weight loss (Chevrier et al. 2000). Thus, we defined effects of PCB-77 administered during the weight gain phase of HF feeding on glucose homeostasis during weight loss. Administration of PCB-77 during the weight gain phase had no effect on body weight reductions during the weight loss phase [see Supplemental Material, Figure S7 (http://dx.doi.org/10.1289/ehp.1205421)]. Glucose tolerance was significantly improved by weight loss in vehicle- or PCB-77–treated mice (week 16 vs. week 12; Figure 3A; *p* < 0.05). Insulin tolerance was also significantly improved by weight loss in vehicle-treated mice (week 16 vs. week 12; Figure 3B; *p* < 0.05). However, compared with controls, mice administered PCB-77 exhibited impaired glucose (Figure 3A; *p* < 0.05) and insulin tolerance (Figure 3B; *p* < 0.05) during the weight loss phase. Weight loss resulted in a significant decrease in *TNF-*α mRNA abundance in adipose tissue from mice administered vehicle or PCB-77 (see Supplemental Material, Figure S8; *p* < 0.05). However, PCB-77–treated mice had significantly increased *TNF-*α mRNA abundance in adipose tissue during weight loss (see Supplemental Material, Figure S8; *p* < 0.05).

*PCB-77 results in an AhR-dependent increase in expression of* TNF-α *in 3T3-L1 adipocytes.* Previous studies demonstrated that PCB-77 increased mRNA abundance of *TNF-*α in 3T3-L1 adipocytes (Arsenescu et al. 2008). TNF-α is an intermediate in reactive oxygen species generated by 2,3,7,8-tetrachloro-dibenzo-*p*-dioxin (TCDD), an AhR ligand (Alsharif et al. 1994). To define mechanisms for effects of coplanar PCBs to promote glucose and insulin intolerance, we examined effects of PCB-77 on *TNF-*α expression in 3T3-L1 adipocytes. Moreover, to determine whether effects of PCB-77 are AhR-mediated, we incubated cells with an AhR antagonist, α-NF. Incubation of differentiated 3T3-L1 adipocytes with PCB-77 significantly increased *CYP1A1* mRNA abundance, indicative of AhR activation [see Supplemental Material, Figure S9A; *p* < 0.05 (http://dx.doi.org/10.1289/ehp.1205421)], which was abolished by α-NF. Similarly, PCB-77-induced increases in *TNF-*α mRNA were abolished by α-NF in 3T3-L1 adipocytes (see Supplemental Material, Figure S9B; *p* < 0.05).

## Discussion

Results from this study indicate that coplanar PCBs induce rapid and sustained glucose and insulin intolerance in lean mice in an AhR-dependent manner. These effects were associated with pronounced accumulation of PCB to adipose tissue, most likely contributing to adipose-specific increases in expression of TNF-α. Remarkably, in PCB-exposed mice that became obese from consumption of a HF diet, the harmful effects of the toxicant promotion of glucose and insulin intolerance were lost. However, when the PCB-exposed obese mice lost weight, glucose and insulin tolerance were impaired, mitigating the beneficial effects of weight loss to improve glucose homeostasis. Moreover, adipose expression levels of *TNF*-α, while not influenced by PCB-77 during the weight gain phase of HF feeding, were increased upon weight loss. In cultured adipocytes, PCB-77 promoted *TNF*-α expression through an AhR-dependent mechanism. These results suggest that PCBs could promote insulin resistance through adipose-specific increases in TNF-α. Moreover, these results suggest that in obese mice with a greater body burden of PCBs, the concentration of PCB was increased in adipose tissue, resulting in increased adipose *TNF*-α expression and insulin resistance upon liberation of PCBs during weight loss.

Increasing evidence suggests that background exposure to persistent organic pollutants is linked to the development of T2D. U.S. Air Force veterans of the Vietnam War who were exposed to Agent Orange contaminated with dioxin had increased risk of diabetes, reduced time-to-onset of disease, and increased diabetes severity (Henriksen et al. 1997). In a cross-sectional study among the general population of Japan and covering the years 2002–2006, blood levels representing the highest quartiles of PCB-126 and PCB-105 had adjusted odds ratios of 9.1 and 7.3, respectively (Uemura et al. 2009). Recent results from the Anniston Community Health Survey demonstrated significant associations between elevated PCB levels and diabetes (Silverstone et al. 2012). Serum levels of PCB-77 in the present study (≈ 3 µM) were similar to levels (1.43 ppb, equivalent to ≈ 5 µM) observed in the lowest quartile of subjects from the Anniston study (Silverstone et al. 2012). Our results demonstrate that lean mice respond to coplanar PCBs with impaired glucose and insulin tolerance, supporting an interaction between PCB exposures and the development of insulin resistance. We used this mouse model of PCB-induced glucose and insulin intolerance to define mechanisms linking PCB exposures to dysregulated glucose homeostasis.

In the present study we demonstrated that PCB-77, a coplanar PCB abundant in the environment (McFarland and Clarke 1989), as well as PCB-126, a coplanar PCB that has been linked to diabetes (Everett et al. 2007), both resulted in dose-dependent rapid impairment of glucose and insulin tolerance in lean mice. Interestingly, mice exposed to the highest dose of either PCB did not have impaired glucose or insulin tolerance; however, these mice demonstrated abnormal behavior (lethargy, tremors), polyuria, and gained minimal weight (unpublished observations), suggesting that higher doses of PCB had deleterious health consequences. Recent studies demonstrated that consumption of farmed salmon containing persistent organic pollutants, including increased levels of seven different PCBs, promoted glucose intolerance associated with elevations in adipose tissue expression of TNF-α in HF-fed mice (Ibrahim et al. 2011). Interestingly, when levels of pollutants were decreased by feeding farm-raised salmon purified fish oil, glucose tolerance improved and adipose expression levels of TNF-α decreased. Our results are in agreement with these findings, and extend these studies by demonstrating that individual coplanar PCBs promote glucose and insulin intolerance associated with adipose-specific elevations in expression of TNF-α. Moreover, similar to previous findings (Ibrahim et al. 2011), adipose contained markedly higher PCB levels than did liver and serum, suggesting that chronic exposures of adipocytes to PCBs most likely contributed to selective increases in TNF-α expression in adipose tissue but not in liver or muscle.

Previous studies in our laboratory demonstrated that coplanar PCBs increased abundance of *TNF*-α mRNA in cultured adipocytes (Arsenescu et al. 2008). Results from the present study demonstrate that PCB-77–induced increases in *TNF*-α expression are AhR mediated. Moreover, concentrations (3.4 µM) of PCB-77 we used in *in vitro* studies with cultured adipocytes were comparable to serum levels (3 µM) of PCB-77 that induced glucose and insulin intolerance in mice. Kim et al. (2012) reported that exposures of 3T3-L1 adipocytes to dioxin increased mRNA abundance of several proinflammatory factors, including TNF-α receptors. In that study, when dioxin was administered to mice fed standard mouse diet, abundance of *TNF-*α mRNA in adipose tissue was markedly increased, and this effect was abolished in AhR deficient mice. Results of the present study confirm and extend previous studies by demonstrating that *in vivo* effects of PCB-77 to impair glucose homeostasis are AhR mediated. An interesting finding of our study was that levels of PCB-77 in adipose tissue were markedly decreased 4 weeks after the last dose; however, mice continued to exhibit glucose and insulin intolerance. PCB-induced activation of AhR induces *CYP1A1* gene expression, which hydroxylates the toxicant to increase water solubility for elimination. Our findings of rapid declines in levels of PCB-77, in the face of sustained impairment of glucose homeostasis, suggest that metabolites of PCB-77 may have contributed to long-lasting impairment of glucose and insulin intolerance in mice.

In this study, although PCB-77 impaired glucose and insulin tolerance in lean mice, these effects were lost when mice were fed a HF diet. The lipophilic nature of PCBs results in their accumulation in adipocyte lipid-containing droplets. For example, in humans, body mass index is inversely correlated with serum levels of PCBs, supporting the concept that an expanded adipose mass results in redistribution of PCBs away from the circulating compartment (Dirinck et al. 2011). In support, Kim et al. (2011) reported an increased (2.9-fold) total body burden of PCBs in obese subjects. Thus, sequestration of PCB-77 in adipocyte lipid pools, as demonstrated by higher levels in adipose tissue from HF-fed mice in the present study, most likely resulted in restricted access to AhR, contributing to a lack of effect of PCB-77 on glucose and/or insulin tolerance in obese mice. Alternatively, effects of PCB-77 to impair glucose homeostasis may have become apparent with longer durations of HF-feeding. Previous studies found that plasma levels of 13 of 17 measured organochlorines in human serum increased with weight loss (Pelletier et al. 2002). Similarly, obese subjects experiencing drastic weight loss from bariatric surgery had increased serum levels of PCBs, which decreased the beneficial effects of weight loss (Kim et al. 2011). In agreement with previous findings, our results demonstrate that beneficial effects of weight loss to improve glucose and insulin tolerance in mice were blunted in mice administered PCB-77. Moreover, our results extend previous findings by showing that elevations in TNF-α in adipose tissue may have contributed to impaired glucose and insulin tolerance in PCB-exposed mice experiencing weight loss.

## Conclusion

Results from this study demonstrate that coplanar PCBs cause rapid and sustained impairment of glucose and insulin tolerance in mice through an AhR-dependent mechanism associated with an adipose-specific increase in TNF-α expression. Although harmful effects of PCB-77 on glucose and insulin tolerance were absent in obese mice, beneficial effects of weight loss to improve glucose and insulin tolerance were mitigated in mice previously exposed to PCB-77. These results suggest that sequestration of lipophilic coplanar PCBs to adipose tissue may contribute to AhR-mediated increases in TNF-α and the development of adipocyte insulin resistance, an effect manifest in lean conditions as well as during weight loss when adipose-derived toxicants may be liberated.

## Supplemental Material

(586 KB) PDF
