# Supplementary material for: Coplanar Polychlorinated Biphenyls Impair Glucose Homeostasis in Lean C57BL/6 Mice and Mitigate Beneficial Effects of Weight Loss on Glucose Homeostasis in Obese Mice
Source: Environ Health Perspect. 2012 Oct 24;121(1):105–10. doi: 10.1289/ehp.1205421 (PMC3553436; doi:10.1289/ehp.1205421)
Supplement: (586 KB) PDF [file ehp.1205421.s001.pdf]

Supplemental Material

Coplanar Polychlorinated Biphenyls Impair Glucose Homeostasis in Lean C57BL/6 Mice and Mitigate Beneficial Effects of Weight Loss on Glucose Homeostasis in Obese Mice

Nicki Baker,[1] Michael Karounos,[1] Victoria English,[1] Jun Fang,[2] Yinan Wei,[2] Arnold Stromberg,[3] Manjula Sunkara,[4] Andrew J. Morris,[4] Hollie I. Swanson,[5] Lisa A. Cassis[1]

Supplemental Material Table of Contents

Item	Page Number
Supplemental Material, Figure S1: Insulin tolerance tests in mice administered vehicle, PCB-77 or PCB-126	2
Supplemental Material, Figure S2: Glucose and insulin tolerance tests in mice administered vehicle, CH-223191, or PCB-77	3
Supplemental Material, Figure S3: PCB-77 results in sustained impairment of glucose and insulin tolerance in LF-fed mice	4
Supplemental Material, Figure S4: PCB-77 increases mRNA abundance of CYP1A1 in adipose and liver of LF-fed mice	5
Supplemental Material, Figure S5:TNF-α and F4/80 mRNA abundance in adipose from mice administered vehicle or PCB-77	6
Supplemental Material, Figure S6: Plasma concentrations of TNF-α and IL-6 following administration of vehicle or PCB-77	7
Supplemental Material, Figure S7: Body weight in LF and HF-fed mice administered vehicle or PCB-77 during the weight gain phase of HF feeding and after mice are switched to a LF diet at week 12 to induce weight loss	8
Supplemental Material, Figure S8: TNF-α mRNA abundance in adipose from mice during weight gain and after mice are switched to a LF diet to induce weight loss	9
Supplemental Material, Figure S9: CYP1A1 and TNF-α mRNA abundance in 3T3-L1 adipocytes incubated with vehicle, α-NF, PCB-77, or PCB-77 + α-NF	10

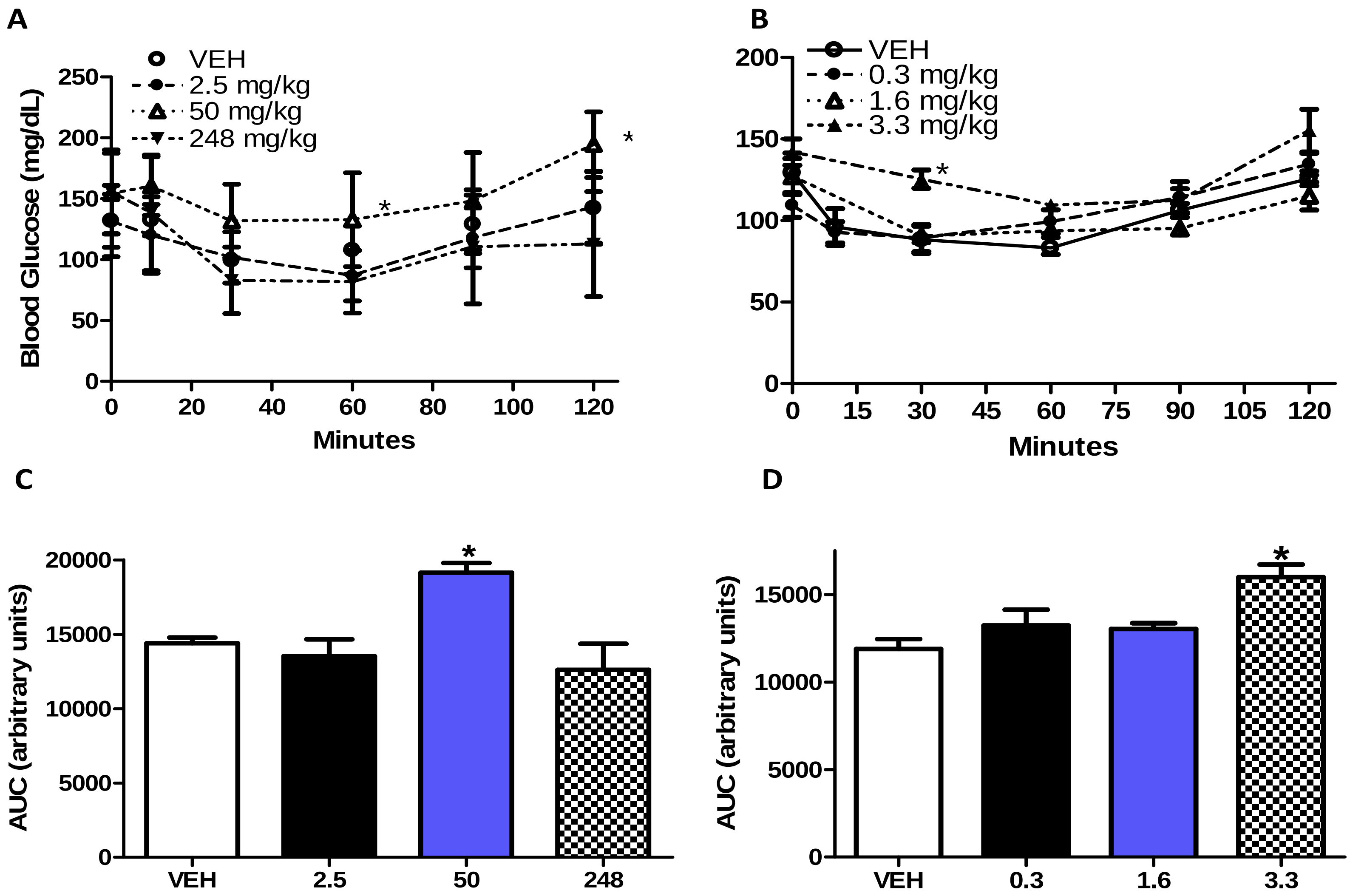

Figure S1. Insulin tolerance tests in mice administered vehicle (VEH), PCB-77 (2.5, 50 or 248 mg/kg, A) or PCB-126 (0.3, 1.6 or 3.3 mg/kg, B). At 48 hours after the last dose, insulin (0.0125 μM/gm body weight) was administered to mice from each treatment group and blood glucose concentrations quantified at several time points. (C, D), Total area under the curve (AUC) for data in A, B, above. Data are mean ± SEM from n = 5 mice/group. *, $P < 0.05$ compared to vehicle.

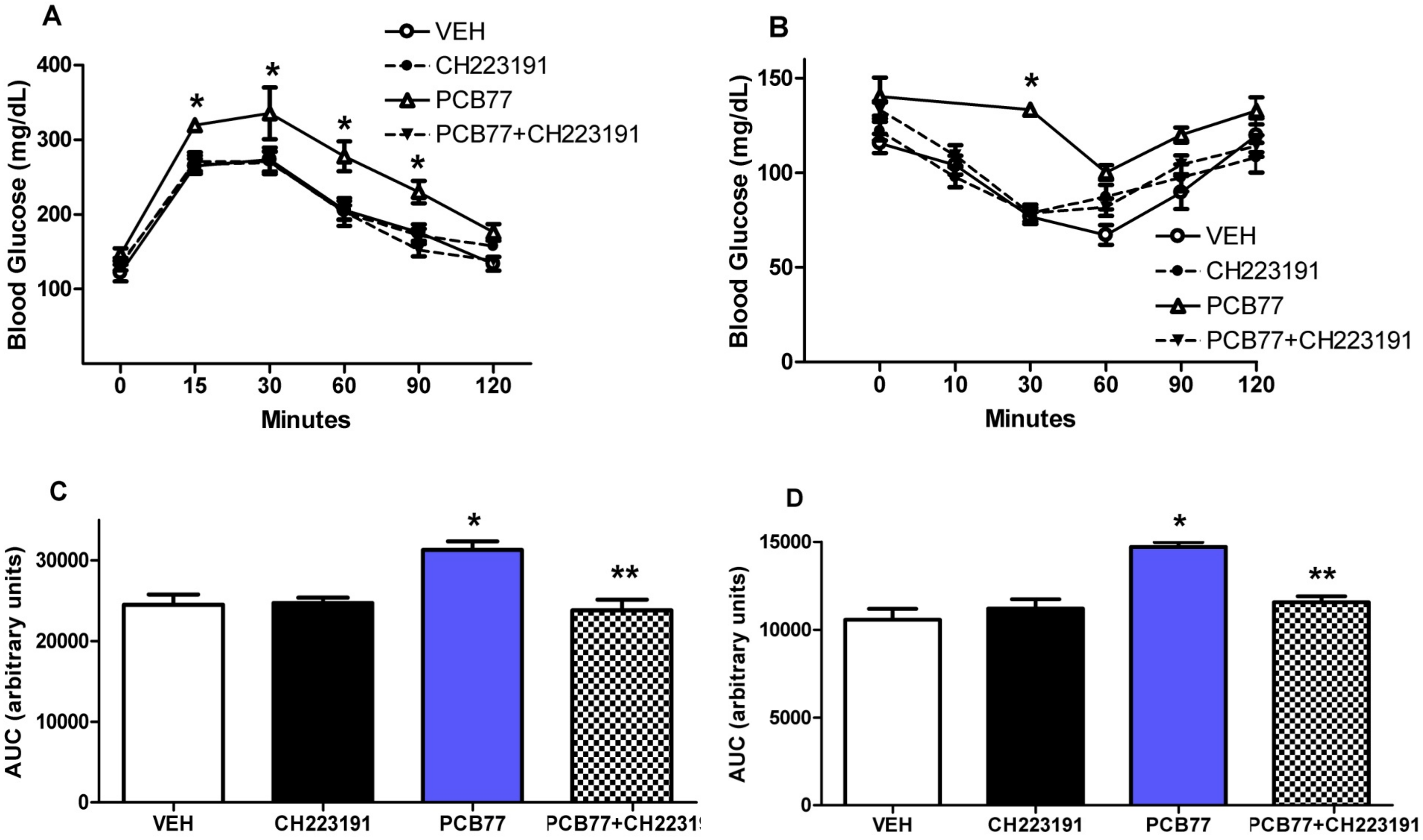

Figure S2. Glucose (A) and insulin (B) tolerance tests in mice administered vehicle (VEH), CH-223191 (10 mg/kg/day), or PCB-77 (50 mg/kg. (C, D) Total area under the curve (AUC) for data in A, B, above. Data are mean ± SEM from n ≥ 7 mice/group. *, $P < 0.05$ compared to vehicle. **, $P < 0.05$ compared to PCB77.

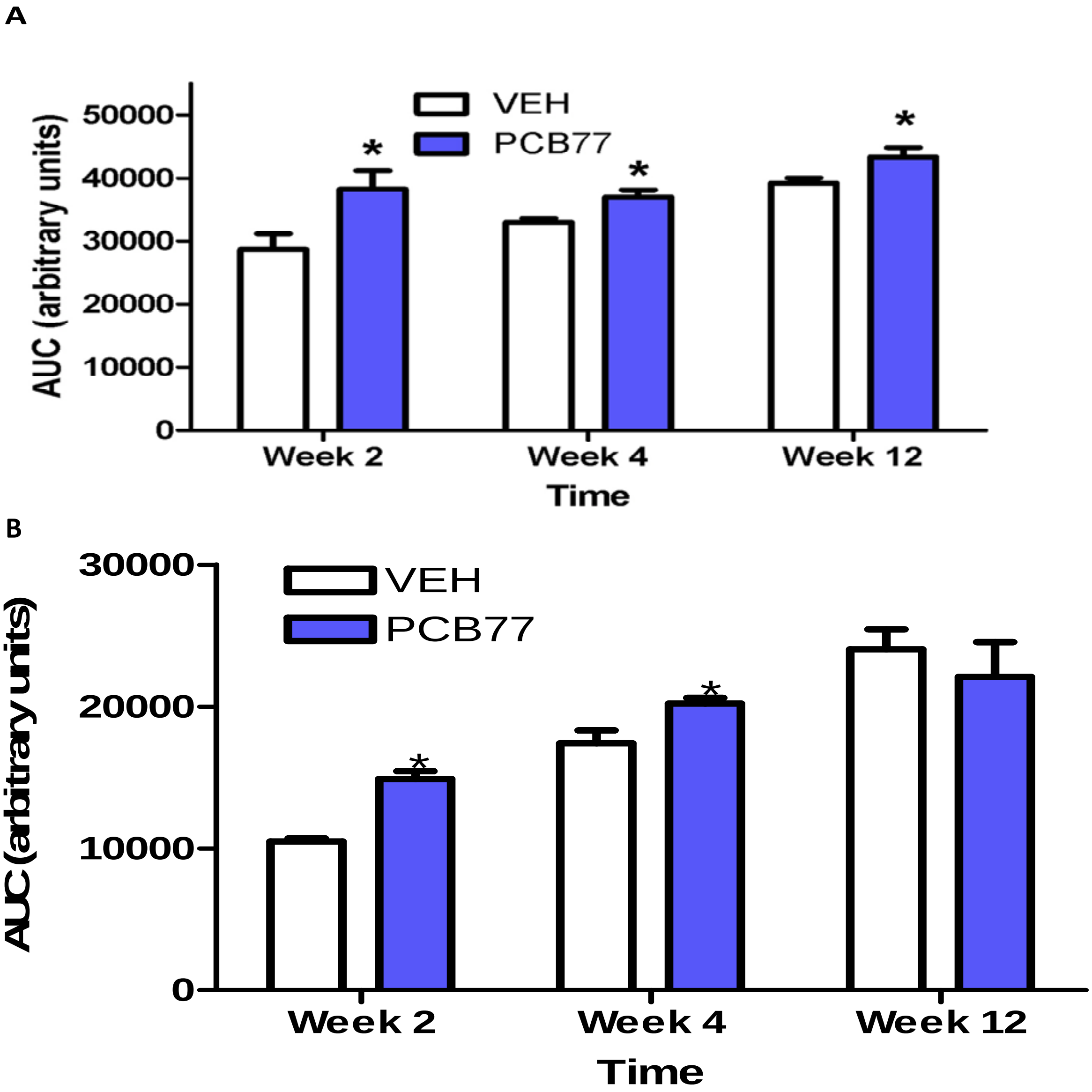

Figure S3. PCB-77 results in sustained impairment of glucose (A) and insulin (B) tolerance in LF-fed mice. Mice were administered vehicle (VEH) or PCB-77 (50 mg/kg, two divided doses during weeks 1 and 2, a second set of 2 doses during weeks 9 and 10 for mice studied at 12 weeks). At 2, 4 or 12 weeks, mice in each treatment group were administered a bolus of glucose (20% glucose, A) or insulin (0.0125 μM/gm body weight, B) and blood glucose concentrations were quantified. Total area under the curve (AUC) for blood glucose concentrations were quantified. Data are mean ± SEM from $n \geq 5$ mice/group. *, $P < 0.05$ compared to VEH within time point.

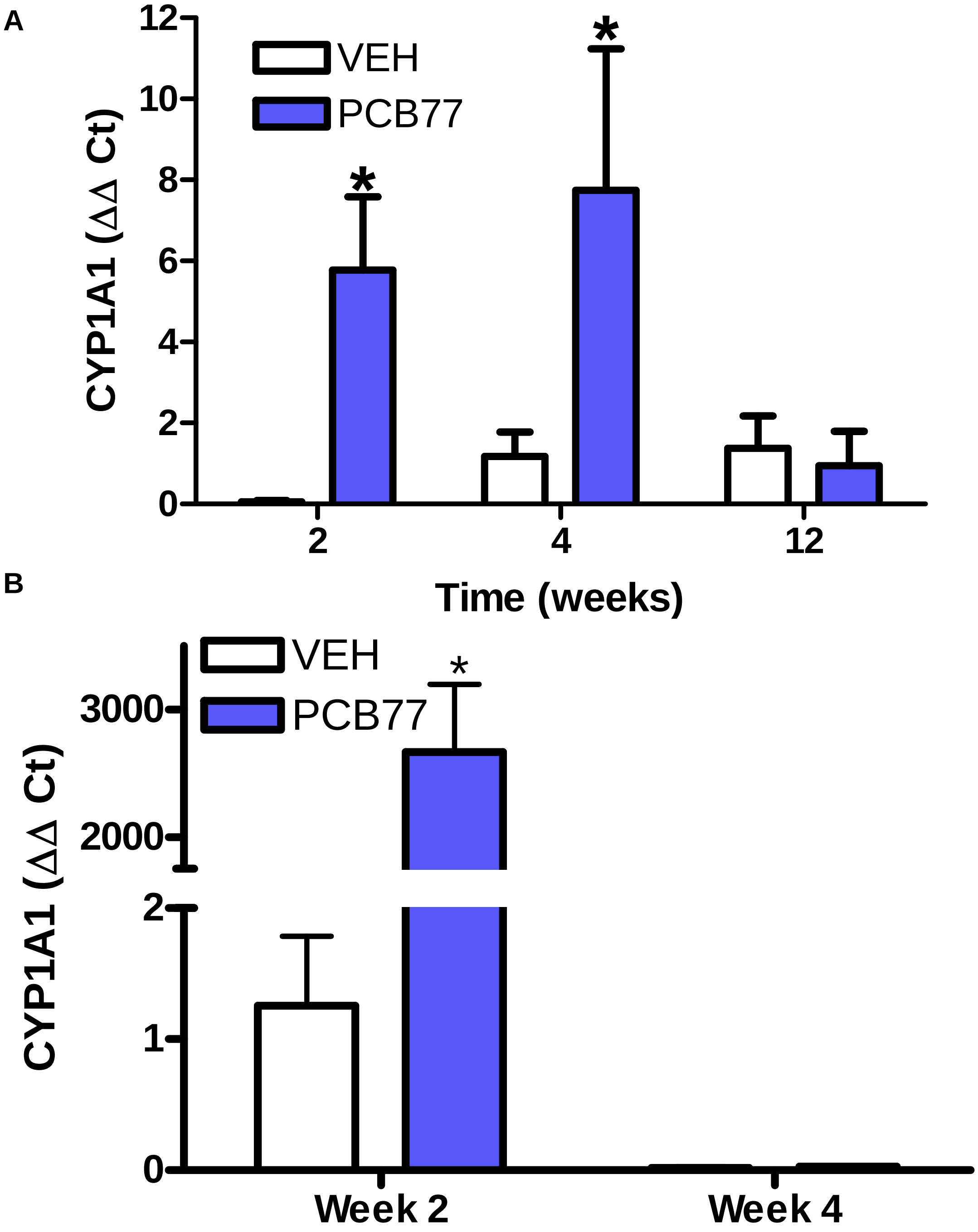

Figure S4. PCB-77 increases mRNA abundance of CYP1A1 in adipose (A) and liver (B) of LF-fed mice. Mice were administered vehicle (VEH) or PCB-77 (50 mg/kg, 2 doses in weeks 1 and 2, second 2 doses in weeks 9 and 10 for 12 week mice). (A), CYP1A1 mRNA abundance was increased in adipose at 1 and 4 weeks, but not 12 weeks after PCB-77 administration. (B), CYP1A1 mRNA abundance was increased in liver at week 2, but not week 4 after PCB-77 administration. Data are mean ± SEM from n ≥ 3 mice/group. *, $P < 0.05$ compared to VEH.

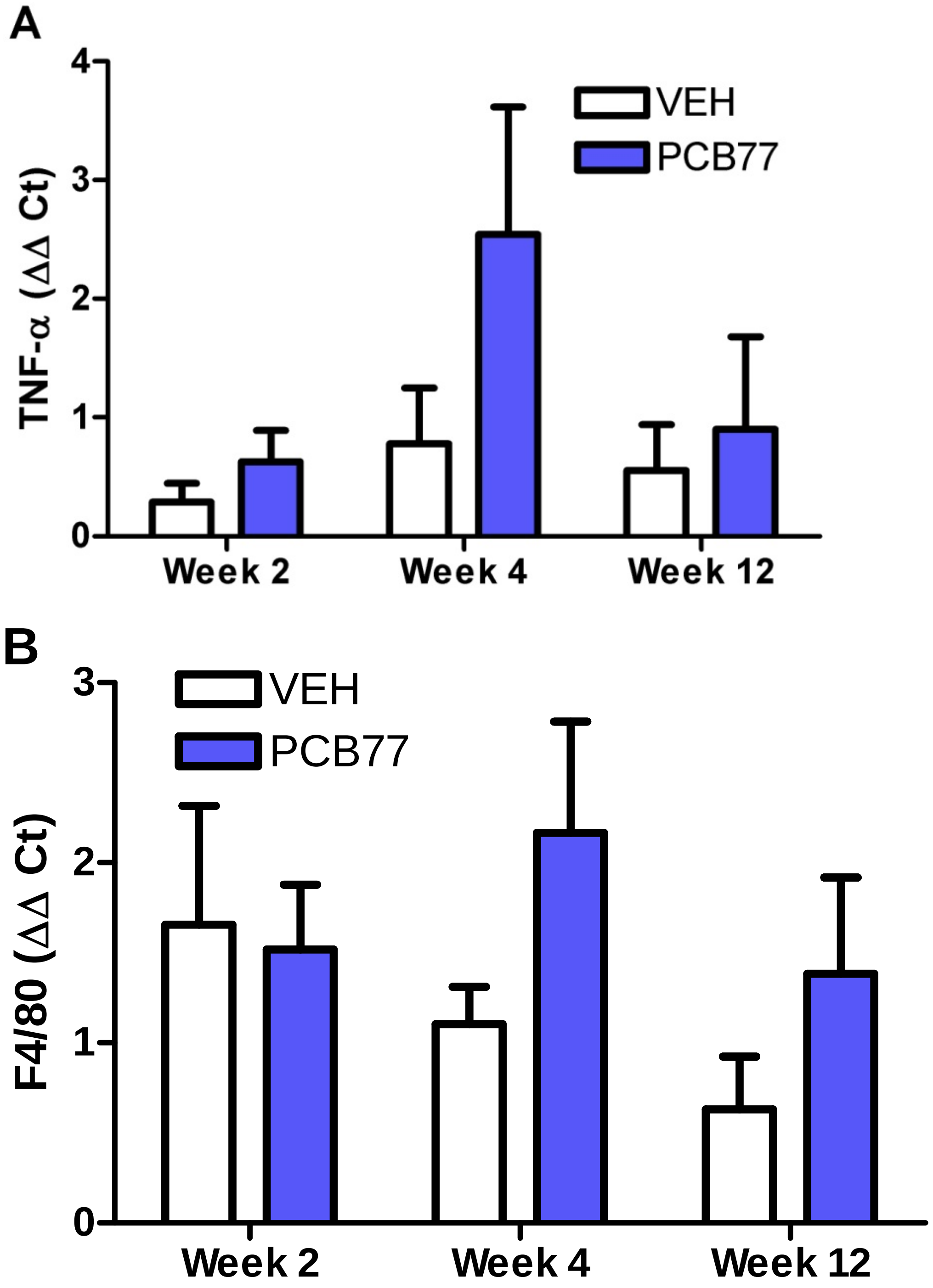

Figure S5. TNF-α (A) and F4/80 (B) mRNA abundance in adipose from mice administered vehicle (VEH) or PCB-77 (50 mg/kg, 2 doses in weeks 1, 2, and then in weeks 9, 10 for mice euthanized at week 12). Data are mean ± SEM from n = 6 mice/group.

A

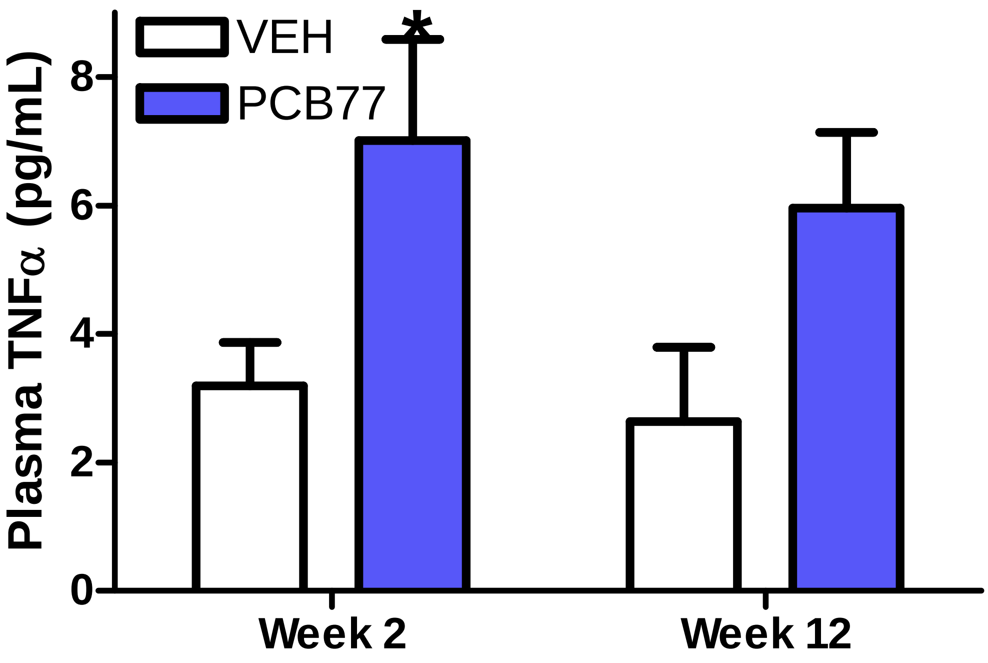

B

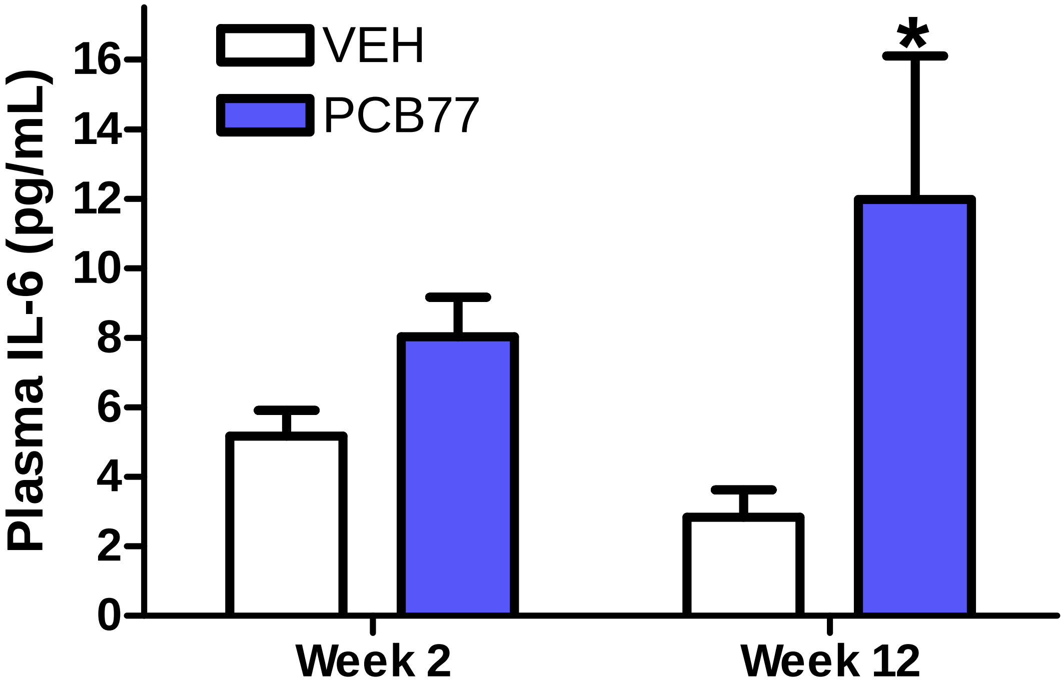

Figure S6. Plasma concentrations of TNF-α (A) and IL-6 (B) following administration of vehicle (VEH) or PCB-77 (50 mg/kg, 2 doses in weeks 1 and 2, second 2 doses in weeks 9 and 10). Data are mean ± SEM from n = 10 mice/group. *, $P < 0.05$ compared to VEH.

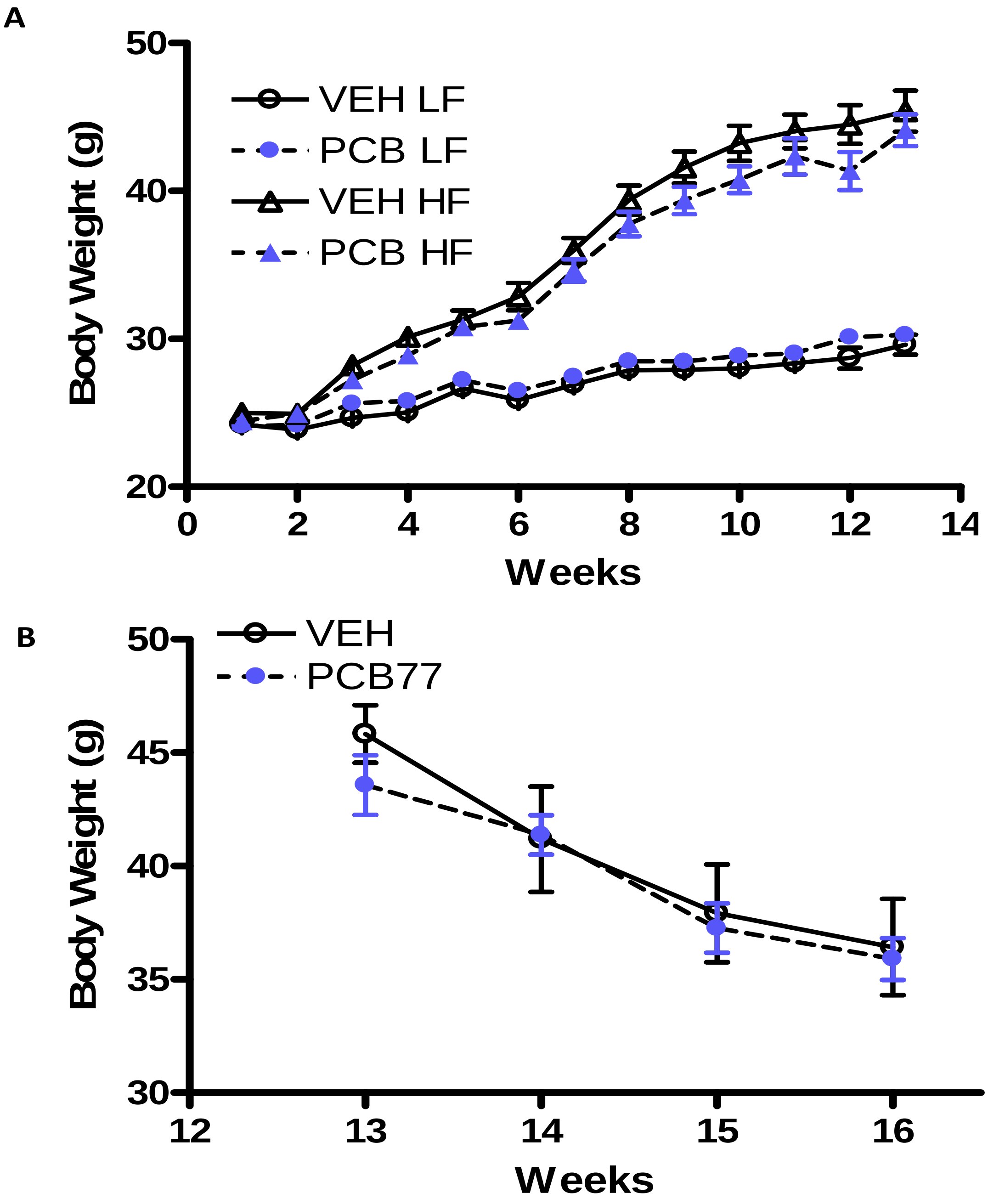

Figure S7. Body weight in LF and HF-fed mice administered vehicle (VEH) or PCB-77 (50 mg/kg, doses in weeks 1, 2, 9, and 10) during the weight gain phase of HF feeding (A), and after mice are switched to a LF diet at week 12 – 16 to induce weight loss (B). Body weight was increased in HF compared to LF-fed mice beginning on week 4 (A). Data are mean ± SEM from n ≥ 5 mice/group.

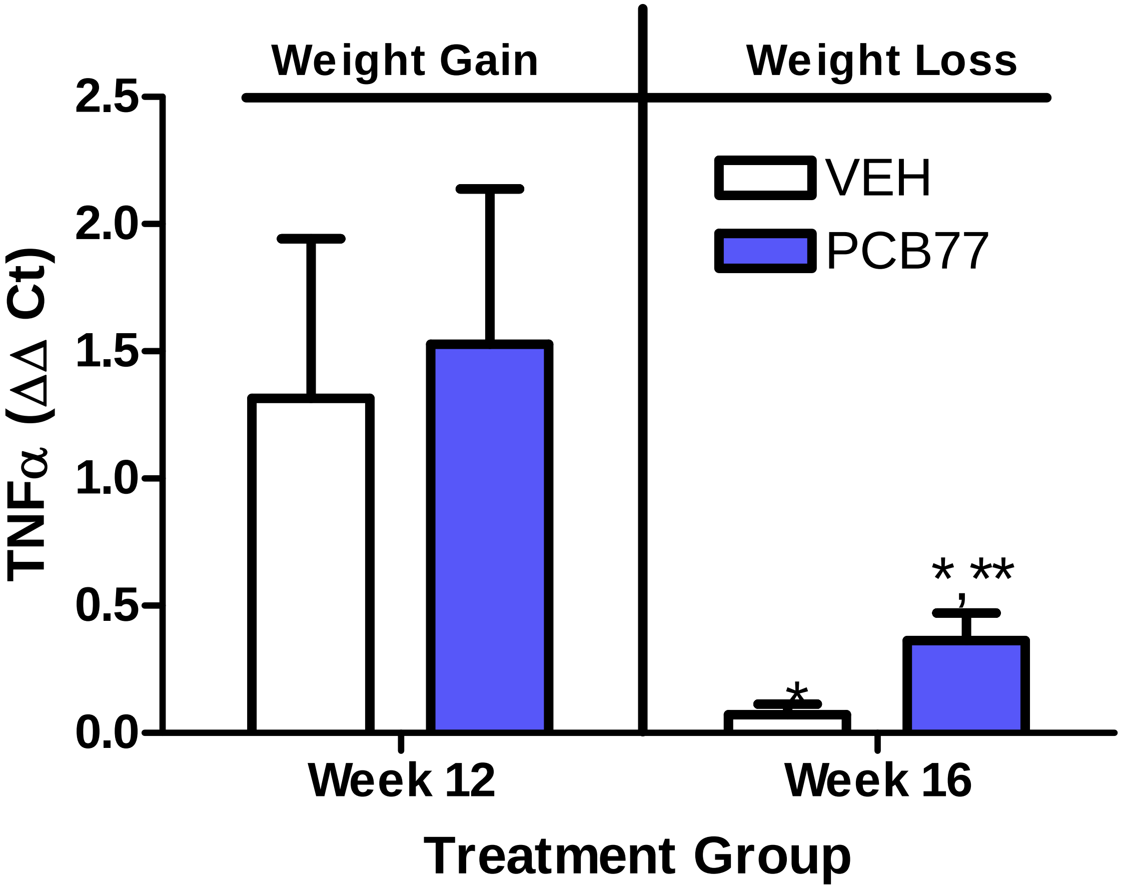

Figure S8. TNF-α mRNA abundance in adipose from mice during weight gain (week 12 of HF feeding), and after mice are switched to a LF diet to induce weight loss (week 16). Data are mean ± SEM from n $\geq$ 3 mice/group. *, $P < 0.05$ compared to week 12 within treatment group. **, $P < 0.05$ compared to VEH within time point.

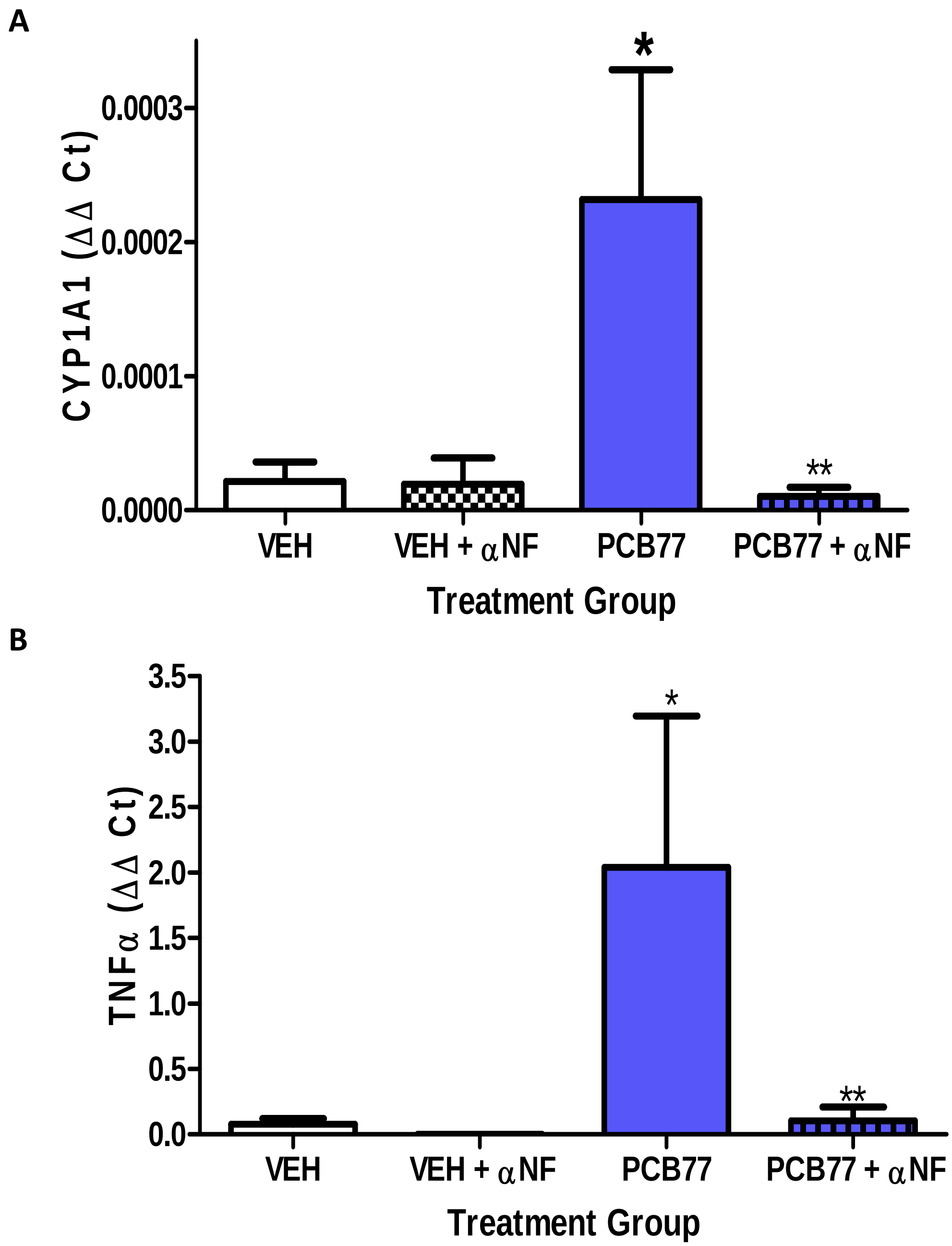

Figure S9

Figure S9. CYP1A1 (A) and TNF-α (B) mRNA abundance in 3T3-L1 adipocytes incubated with vehicle, α-NF, PCB-77, or PCB-77 + α-NF. Data are mean ± SEM from n = 3 experiments. *, $P < 0.05$ compared to vehicle. **, $P < 0.05$ compared to PCB-77.
